# Photosynthesis of C_3_, C_3_–C_4_, and C_4_ grasses at glacial CO_2_

**DOI:** 10.1093/jxb/eru155

**Published:** 2014-04-10

**Authors:** Harshini Pinto, Robert E. Sharwood, David T. Tissue, Oula Ghannoum

**Affiliations:** Hawkesbury Institute for the Environment, University of Western Sydney, Hawkesbury campus, Locked Bag 1797, Penrith 2751, NSW, Australia

**Keywords:** C_3_, C_3_–C_4_, and C_4_ photosynthesis, glacial CO_2_, NAD-ME, NADP-ME, PEPC, PEP-CK, Rubisco, water and nitrogen use efficiency.

## Abstract

Most physiology comparisons of C_3_ and C_4_ plants are made under current or elevated concentrations of atmospheric CO_2_ which do not reflect the low CO_2_ environment under which C_4_ photosynthesis has evolved. Accordingly, photosynthetic nitrogen (PNUE) and water (PWUE) use efficiency, and the activity of the photosynthetic carboxylases [Rubisco and phosphoenolpyruvate carboxylase (PEPC)] and decarboxylases [NADP-malic enzyme (NADP-ME) and phosphoenolpyruvate carboxykinase (PEP-CK)] were compared in eight C_4_ grasses with NAD-ME, PCK, and NADP-ME subtypes, one C_3_ grass, and one C_3_–C_4_ grass grown under ambient (400 μl l^–1^) and glacial (180 μl l^–1^) CO_2_. Glacial CO_2_ caused a smaller reduction of photosynthesis and a greater increase of stomatal conductance in C_4_ relative to C_3_ and C_3_–C_4_ species. *Panicum bisulcatum* (C_3_) acclimated to glacial [CO_2_] by doubling Rubisco activity, while Rubisco was unchanged in *Panicum milioides* (C_3_–C_4_), possibly due to its high leaf N and Rubisco contents. Glacial CO_2_ up-regulated Rubisco and PEPC activities in concert for several C_4_ grasses, while NADP-ME and PEP-CK activities were unchanged, reflecting the high control exerted by the carboxylases relative to the decarboxylases on the efficiency of C_4_ metabolism. Despite having larger stomatal conductance at glacial CO_2_, C_4_ species maintained greater PWUE and PNUE relative to C_3_–C_4_ and C_3_ species due to higher photosynthetic rates. Relative to other C_4_ subtypes, NAD-ME and PEP-CK grasses had the highest PWUE and PNUE, respectively; relative to C_3_, the C_3_–C_4_ grass had higher PWUE and similar PNUE at glacial CO_2_. Biomass accumulation was reduced by glacial CO_2_ in the C_3_ grass relative to the C_3_–C_4_ grass, while biomass was less reduced in NAD-ME grasses compared with NADP-ME and PCK grasses. Under glacial CO_2_, high resource use efficiency offers a key evolutionary advantage for the transition from C_3_ to C_4_ photosynthesis in water- and nutrient-limited environments.

## Summary

At glacial CO_2_, NAD-ME grasses have higher photosynthetic water use efficiency than NADP-ME and PCK counterparts. Photosynthetic carboxylases rather than decarboxylases modulate the response of C_4_ photosynthesis to glacial CO_2_

## Introduction

The decline in atmospheric CO_2_ concentration ([CO_2_]) in the late Oligocene (30 million years ago) is considered to be the primary driver for the evolution of the C_4_ photosynthetic pathway (Christin *et al.*, 2008; Ehleringer *et al.*, 1997; Sage *et al.*, 2012). Geological fluctuations in atmospheric [CO_2_] have shaped the Earth’s vegetation, yet relatively little is known about the responses of C_4_ plants to the low [CO_2_] levels that dominated during their evolution, and that are close to the atmospheric [CO_2_] of the recent glaciation (Pagani *et al.*, 2005). Low [CO_2_] promotes high rates of photorespiration and reduces the carboxylation efficiency of C_3_ photosynthesis. The key feature of C_4_ photosynthesis is the operation of a CO_2_-concentrating mechanism (CCM) which suppresses photorespiration by raising [CO_2_] around Rubisco (ribulose-1,5-bisphosphate carboxylase/oxygenase). During C_4_ photosynthesis, phosphoenolpyruvate carboxylase (PEPC) catalyses the initial carboxylation of CO_2_ into organic C_4_ acids in the mesophyll. Decarboxylation of C_4_ acids in the bundle sheath releases CO_2_ for refixation by Rubisco (Hatch, 1987). The C_4_ photosynthetic pathway is classified into three biochemical subtypes based on the primary C_4_ decarboxylase enzyme. These enzymes are NADP-malic enzyme (NADP-ME), NAD-malic enzyme (NAD-ME), and phosphoenolpyruvate carboxykinase (PEP-CK, also known as PCK) (Gutierrez *et al.*, 1974; Kanai and Edwards, 1999). There are strong anatomical and biochemical variations associated with these biochemical subtypes (Prendergast *et al.*, 1987; Dengler *et al.*, 1994; Edwards and Voznesenskaya, 2011).

The operation of a CCM enhances the efficiency of C_4_ relative to C_3_ photosynthesis (Osmond, 1982). In particular, C_4_ species attain higher photosynthetic water use efficiency (PWUE) because lower stomatal conductance (*g*_s_) and intercellular [CO_2_] (*C*_i_) are needed to saturate Rubisco carboxylation. C_4_ plants achieve higher photosynthetic nitrogen use efficiency (PNUE) due to their lower leaf N requirement as a result of a higher Rubisco catalytic turnover rate (*k*_cat_) (Long, 1999; Taylor *et al.*, 2010; Ghannoum *et al.*, 2011). Variations in resource use efficiency also occur among the C_4_ subtypes (Ghannoum *et al.*, 2011). For example, NADP-ME grasses tend to have lower leaf N content than their NAD-ME counterparts (Bowman, 1991; Knapp and Medina, 1999; Taub and Lerdau, 2000), as a result of faster Rubisco *k*_cat_ in NADP-ME species (Ghannoum *et al.*, 2005). Furthermore, Ghannoum *et al.* (2002) showed that under water stress, NAD-ME grasses increased their whole-plant WUE to a greater extent than NADP-ME counterparts. These aforementioned studies were undertaken under current ambient [CO_2_] which does not reflect the low CO_2_ environment under which C_4_ grasses have evolved. Hence, the main aim of the current study was to investigate whether previously reported physiological differences among the C_4_ subtypes at ambient [CO_2_] are similarly observed at glacial [CO_2_].

Growth at low [CO_2_] reduces growth and photosynthesis of C_3_ plants. C_3_ plants respond to low [CO_2_] by increasing *g*_s_ to improve CO_2_ supply and by up-regulating photosynthetic enzymes to improve CO_2_ capture (Polley *et al.*, 1992; Dippery *et al.*, 1995; Tissue *et al.*, 1995; Gesch *et al.*, 2000; Anderson *et al.*, 2001). The occurrence of a CCM in C_4_ leaves makes the C_4_ pathway less limited by CO_2_ supply and, hence, less likely to respond and acclimate to growth at low [CO_2_] relative to C_3_ photosynthesis (Hatch, 1987; Gerhart and Ward, 2010). Nevertheless, increased leaf N content and *g*_s_ have been observed under low [CO_2_] in some C_4_ species (Anderson *et al.*, 2001; Maherali *et al.*, 2002). To the authors’ knowledge there are no published studies comparing the impact of low [CO_2_] on the photosynthetic gas exchange or biochemistry of C_4_ grasses with different biochemical subtypes. The current study aims at addressing this knowledge gap.

A hypothezised intermediate stage during C_4_ evolution, known as C_3_–C_4_ intermediate, restricts the activity of glycine decarboxylase to the bundle sheath (Sage *et al.*, 2012), thus improving Rubisco efficiency by facilitating the recapture of photorespired CO_2_ (Monson and Moore, 1989; Monson and Rawsthorne, 2000). The operation of a photorespiratory pump in C_3_–C_4_ photosynthesis is expected to elicit a response to [CO_2_] that is intermediate between C_3_ and C_4_ photosynthesis (Monson and Rawsthorne, 2000; Sage *et al.*, 2012). Under low [CO_2_], C_3_–C_4_ plants have been reported to maintain greater photosynthetic rates, PWUE, and PNUE relative to C_3_ species (Ku and Edwards, 1978; Bolton and Brown, 1980; Ku *et al.*, 1991; Monson and Rawsthorne, 2000; Vogan *et al.*, 2007; Pinto *et al.*, 2011; Vogan and Sage, 2012). The current study seeks to determine how C_3_–C_4_ species perform relative to the various C_4_ subtypes at low [CO_2_].

Comparing the sensitivity to glacial [CO_2_] of the different pathways of photosynthesis and subtypes of C_4_ photosynthesis among closely related grass species may provide critical insight into the physiology of C_4_ plants under conditions that led to their evolution. Consequently, this study compared the photosynthetic physiology (PWUE and PNUE) and biochemistry (activity of the photosynthetic carboxylase and decarboxylase enzymes) in C_4_ grasses with different biochemical subtypes grown under ambient (400 μl l^–1^) or glacial (180 μl l^–1^) [CO_2_]. Closely related C_3_ and C_3_–C_4_ grass species were included for comparison.

## Materials and methods

### Plant culture

Two matched growth chambers (1.8 m^3^ each; BioChambers, Winnipeg, Manitoba, Canada) were used in this study. The chambers were maintained at either glacial (180 μl l^–1^) or ambient (400 μl l^–1^) [CO_2_]. Low [CO_2_] was achieved by passing incoming air over a CO_2_ absorbent (Grace SodaSorb, WR Grace and Co.-Conn., Chicago, USA) and controlled by CO_2_ gas analysers (LI-820, LI-COR, Lincoln, NE, USA). The average growth conditions during the experiment are shown in Table 1.

**Table 1.** Average growth conditions in the glacial and ambient CO_2_ growth chambers during the experimental period Light intensity was measured at the pot level. The photoperiod was 12h. Values are averages (± standard deviation) over the growing period.

	Glacial CO_2_		Ambient CO_2_	
	Day	Night	Day	Night
Light (μmol m^–2^ s^–1^)	900±2		900±3	
[CO_2_] (μl l^–1^)	181±4	182±2	400±2	400±2
Temperature (°C)	27±1	17±1	27±1	17±1
Relative humidity (%)	70±1	70±1	70±1	70±1

Locally collected soil (Ghannoum *et al.*, 2010) was air-dried, coarsely sieved, and added (3.7kg) to 3.5 l cylindrical pots, which were watered to 100% capacity, then transferred to the two growth chambers. Seeds for the grass species used in this study (Table 2) were obtained from the Australian Plant Genetic Resources Information System (ACT, Australia) and Queensland Agricultural Seeds Pty. Ltd (Toowoomba, Australia). Seeds were sown in trays containing a common germination mix. Three to four weeks after germination, three seedlings were transplanted into each of the soil-filled pots. Within a week of transplanting, one healthy seedling was left in the pot while the other seedlings were removed; there were four pots per species and CO_2_ treatment. Two environmentally controlled growth chambers were used to generate the CO_2_ treatments. In order to minimize the impact of having a single growth chamber per CO_2_ treatment, pots and CO_2_ treatments were switched between chambers on two occasions. In addition, pots were randomly rotated within each chamber on a weekly basis throughout the experiment. Plants were watered daily and a commercial fertilizer (General Purpose, Thrive Professional, Yates, Australia) was applied weekly (0.2g N l^–1^).

**Table 2.** List of grass species used in the current study

Species	Photosynthetic type
*Panicum bisulcatum* Thunb.	C_3_
*Panicum milioides* Nees	C_3_–C_4_
*Astrebla lappacea* (Lindl.) Domin.	C_4_, NAD-ME
*Panicum coloratum* L.	C_4_, NAD-ME
*Heteropogon contortus* (L) P. Beauv. Ex Roem. & Schult.	C_4_, PCK
*Panicum monticola* Hook. F.	C_4_, PCK
*Panicum maximum* Jacq.	C_4_, PCK
*Chloris gayana* Kunth.	C_4_, PCK
*Zea mays* L.	C_4_, NADP-ME
*Echinochloa frumentaceae* L.	C_4_, NADP-ME

### Leaf gas exchange measurements

Gas exchange measurements were made using a portable open gas exchange system (LI-6400XT, LI-COR). At 7–8 weeks after transplanting, gas exchange measurements were made at a photosynthetic photon flux density of 1800 μmol m^–2^ s^–1^ between 10:00h and 14:00h on attached, last fully expanded leaves (LFELs) of the main stems. Spot measurements of the light-saturated photosynthetic rate (*A*_sat_) and *g*_s_ were made at target growth [CO_2_] (180 μl l^–1^ or 400 μl l^–1^) and leaf temperature of 27 ºC. Leaf-to-air vapour pressure deficit ranged between 1.7 kPa and 2.4 kPa during the measurements. Before each measurement, the leaf was allowed to stabilize for 10–20min until it reached a steady state of CO_2_ uptake.

The responses of CO_2_ assimilation rates (*A*) to step increases of *C*_i_ were measured under conditions similar to spot measurements by raising the cuvette [CO_2_] in 10 steps between 50 μl l^–1^ and 1500 μl l^–1^. There were 3–4 replicates per treatment. The *A*–*C*_i_ curves were fitted using the C_4_ photosynthesis model (von Caemmerer, 2000) to estimate maximal PEPC (*in vivo V*_pmax_) and Rubisco (*in vivo V*_cmax_) activities. The biochemical model of C_3_ photosynthesis was used to estimate *V*_cmax_ (apparent, maximal RuBP-carboxylation limited rate) for the C_3_ grass (Farquhar *et al.*, 1980), using Rubisco catalytic parameters obtained for *Panicum bisulcatum* (RE Sharwood, O Ghannoum, and SM Whitney, unpublished).

### Growth and nitrogen analyses

Plants were harvested 12–13 weeks after transplanting. At harvest, the area of the LFELs and total leaf area were measured using a leaf area meter (LI-3100A, LI-COR). Shoots were separated into stems and leaves. Roots were washed free of soil. Plant materials were oven-dried at 80 ºC for 48h before dry mass was measured. Leaf mass per area (LMA, g m^–2^) was calculated as total leaf dry mass/total leaf area. For each treatment, three dried LFELs of each species were milled to a fine powder. Tissue N was determined on the ground samples using a CHN analyser (LECO TruSpec, LECO Corporation, MI, USA).

### Activity of Rubisco, PEPC, NADP-ME, and PEP-CK

Following gas exchange measurements made at growth [CO_2_], replicate leaf discs (1–2cm^2^) were cut under high light and rapidly frozen in liquid nitrogen then stored at –80 °C for biochemical analysis. Each leaf disc was extracted in 1ml of ice-cold extraction buffer [50mM EPPS-NaOH pH 8.0, 5mM dithiothreitol (DTT), 15mM NaHCO_3_, 20mM MgCl_2_, 2mM EDTA, 4% (v/v) protease inhibitor cocktail (Sigma), and 1% (w/v) polyvinylpolypyrrolidone (PVPP)] using a 2ml Potter–Elvehjem glass homogenizer kept on ice. Subsamples (75 μl) were taken from the total extract for SDS–PAGE analysis of total leaf protein. The remaining extract was centrifuged at 16 100 *g* for 1min and the supernatant used for enzyme activity, Rubisco content, and soluble protein assays. Rubisco content was estimated by the irreversible binding of [^14^C]carboxyarabinitol bisphosphate (CABP) to the fully carbamylated enzyme (Ruuska *et al.*, 1998). Rubisco activity (*in vitro V*_cmax_) was estimated by multiplying the concentration of active sites determined using the [^14^C]CABP assay by the *in vitro* turnover rate (*k*_cat_ at 25 °C) of the various C_4_ grasses (Supplementary Table S1 available at *JXB* online). Activities of PEPC and NADP-ME enzymes were determined at 25 °C using a UV-VIS spectrophotometer (model 8453, Agilent Technologies Australia, Mulgrave, Victoria) as previously described by Pengelly *et al.* (2012) and Ashton *et al.* (1990). Soluble proteins were measured using the Pierce Coomassie Plus (Bradford) protein assay kit (Thermo Scientific, Rockford, IL, USA).

PEP-CK activity was assayed at 25 °C in the carboxylase direction (Walker *et al.*, 2002). Each leaf disc was extracted in 1ml of ice-cold extraction buffer [50mM HEPES pH 7.2, 5mM DTT, 2mM EDTA, 2mM MnCl_2_, 0.05% Triton, 4% (v/v) protease inhibitor cocktail (Sigma), and 1% (w/v) PVPP] using a 2ml Potter–Elvehjem glass homogenizer kept on ice. The extract was centrifuged at 16 100 *g* for 1min and the supernatant was used. PEP-CK activity was measured in assay buffer containing 100mM HEPES, pH 7.0, 4% mercaptoethanol (w/v), 100mM KCl, 90mM NaHCO_3_, 1mM ADP, 2mM MnCl_2_, 0.14mM NADH, and malate dehydrogenase after the addition of phosphoenolpyruvate (PEP) to 5mM. It was not possible to assay reliably for NAD-ME activity in this study.

### Immunoblot analysis

To confirm the presence or absence of assayed enzyme activities, especially the decarboxylases in the C_4_ species and PEPC in C_3_ and C_3_–C_4_ species, immunoblot analysis of the proteins in question was carried out. Subsamples of total leaf extracts (used for enzyme assays) were mixed with 0.25vol of 4× LDS buffer (Invitrogen) containing 100mM DTT, snap-frozen in liquid nitrogen, and stored at –20 °C until analysed. Protein samples were separated by SDS–PAGE at 200V using TGX Any kD (Bio-Rad Laboratories, Hercules, CA, USA) pre-cast polyacrylamide gels in the Mini-Protean apparatus buffered with TRIS-glycine SDS buffer (Bio-Rad). Separated proteins were transferred at 4 °C to nitrocellulose membranes (0.45 μm; Bio-Rad) using the Xcell Surelock western transfer module (Invitrogen) buffered with 1× Transfer buffer [20×; 25mM Bicine, 25mM Bis-Tris, 1mM EDTA, 20% (v/v) methanol]. After 1h of transfer at 30V, the membrane was placed in blocking solution [3% (w/v) skim milk powder in TRIS-buffered saline (TBS); 50mM TRIS-HCl pH 8, 150mM NaCl] for 1h at room temperature with gentle agitation.

For immunoblot analysis, primary antisera raised in rabbit against tobacco Rubisco (prepared by SM Whitney) were diluted 1:4000 in TBS before incubation at 1h with membranes at room temperature with gentle agitation. Antiserum raised against PEPC (Cat. AS09 458) was obtained from Agrisera (Agrisera AB, Vännäs, Sweden) and diluted 1:2000 with TBS. For immunoblot analysis of NADP-ME and PEP-CK, synthetic peptides based on monocot amino acid sequences for each were synthesized by GL Biochem [GL Biochem (Shanghai) Ltd., Shanghai, China] and antiserum was raised to each peptide in rabbits. The reactive antisera were antigen purified and used for immunoblots (GL Biochem). The NADP-ME (Product ID A-003198) and PEP-CK (Product ID A-003200) antisera were diluted in TBS 1:1000 and 1:500, respectively. All primary antisera were incubated with membranes at room temperature for 1h with gentle agitation before washing three times with TBS. Secondary goat anti-rabbit antisera conjugated to horseradish peroxidase (HRP; Cat. NEF 812001EA, Perkin Elmer) were diluted 1:3000 in TBS and incubated with the membranes for 1h at room temperature followed by three washes with TBS. Immunoreactive peptides were detected using the Immun-Star Western C kit (Cat. 170–5070, Bio-Rad) and imaged using the VersaDoc imaging system (Bio-Rad).

### Statistical and data analysis

PWUE was calculated as *A*_sat_ (μmol m^–2^ s^–1^)/*g*_s_ (mol m^–2^ s^–1^). PNUE was calculated as *A*_sat_ (μmol m^–2^ s^–1^)/leaf [N]_area_ (mmol m^–2^). The proportion of leaf N invested in Rubisco (Rubisco-N) was calculated by assuming that Rubisco contained 16% N on a mass basis (Evans and Seemann, 1989).

There were four replicates per treatment for growth, gas exchange, and enzyme assay measurements. There were three replicate measurements for the leaf N analysis and the *A*–*C*_i_ curves. The relationship between the various response variables and the main effects (species, photosynthetic type, and CO_2_ treatment) and their interactions were fitted using a linear model in R (V. 3.0.2; R Foundation for statistical computing, Vienna, Austria). Analysis of variance (ANOVA; summarized in Table 2) was conducted for each fitted model. Multiple comparisons (shown in Table 4 and Supplementary Table S1 at *JXB* online) of species means were made using the Tukey test.

## Results

### Photosynthetic rates and WUE

**Fig. 1.**
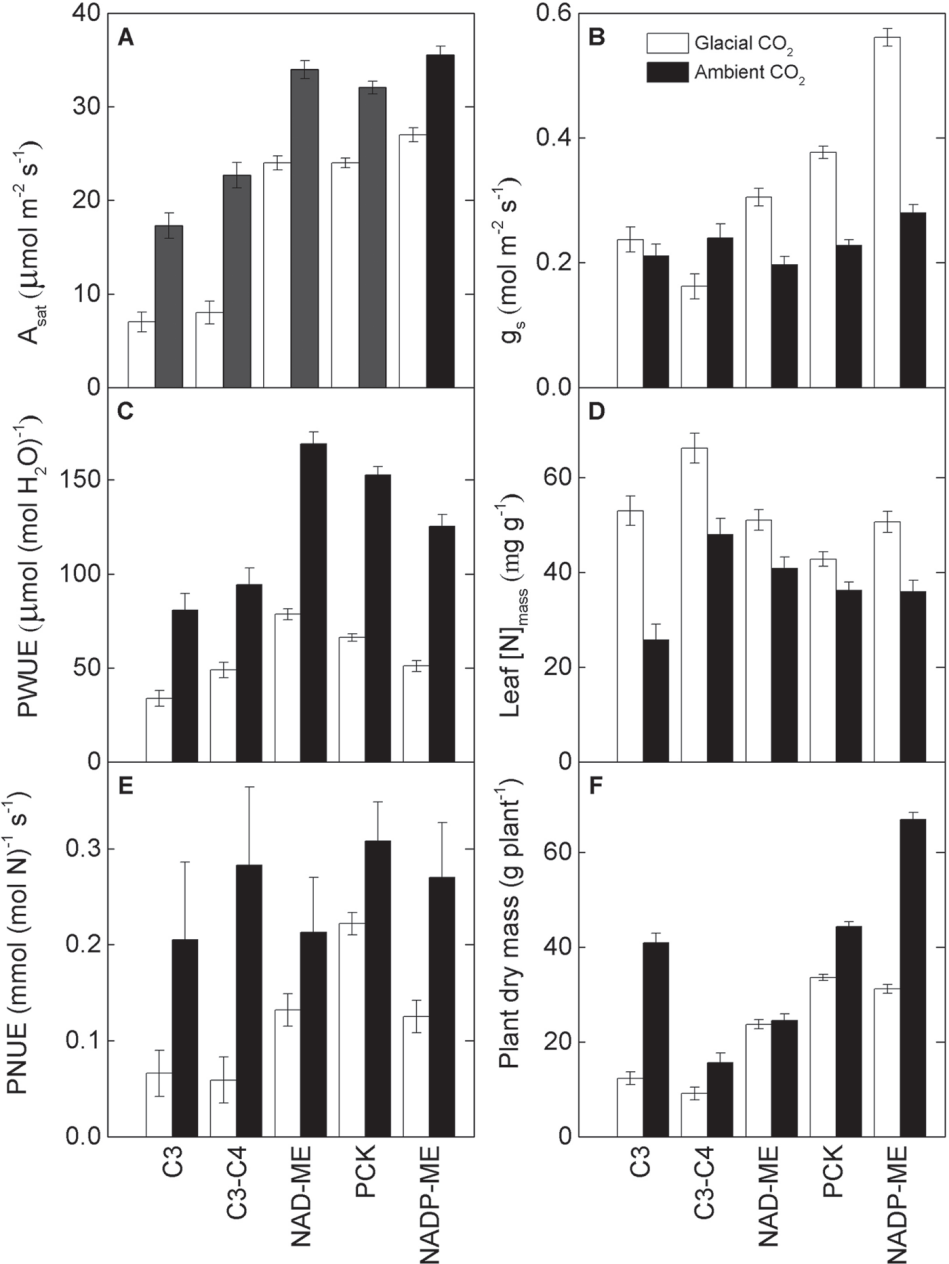
Gas exchange and growth parameters. Light-saturated photosynthesis, *A*_*sat*_ (A), stomatal conductance, *g*_s_ (B), photosynthetic water use efficiency, PWUE (C), leaf N per unit dry mass, [N]_mass_ (D), photosynthetic nitrogen use efficiency, PNUE (E), and plant dry mass, PDM (F) of 10 grass species belonging to C_3_, C_3_–C_4_, and C_4_ (NAD-ME, PCK, NADP-ME) photosynthetic types grown at glacial (180 μl l^–1^, open columns) or ambient (400 μl L^–1^, filled columns) [CO_2_]. Values are means ±SE of species within each photosynthetic type.

**Fig. 2.**
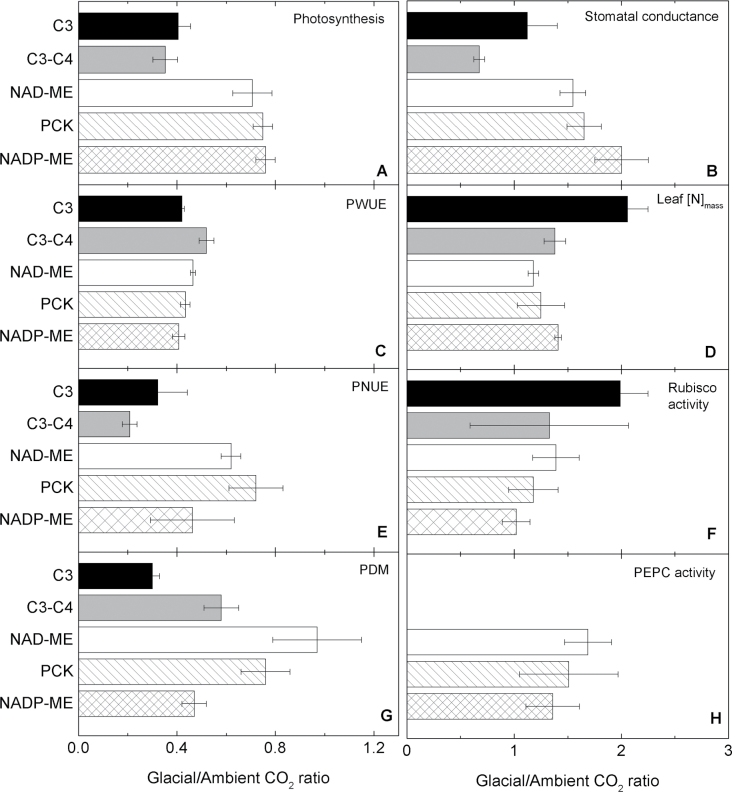
CO_2_ sensitivity of photosynthetic and growth parameters. Glacial to ambient CO_2_ ratios of light-saturated photosynthesis, *A*_sat_ (A), stomatal conductance, *g*_s_ (B), photosynthetic water use efficiency, PWUE (C), leaf N per unit dry mass, [N]_mass_ (D), photosynthetic nitrogen use efficiency, PNUE (E), Rubisco activity (F), plant dry mass, PDM (G), and PEPC activity (H). Original data are shown in Supplementary Table S1 at *JXB* online.

Under both [CO_2_] treatments, photosynthetic rates measured at high light and growth [CO_2_] (*A*_sat_) were higher in the C_4_ species relative to the C_3_–C_4_ and C_3_ species. Amongst the C_4_ species, variation in *A*_sat_ was unrelated to their subtype. Relative to ambient [CO_2_], glacial [CO_2_] decreased *A*_sat_ to a greater extent in the C_3_–C_4_ (65%) and C_3_ (60%) species relative to the C_4_ species (26%) (Figs 1A, 2A; Table 3; Supplementary Table S1 at *JXB* online).

**Table 3.** Statistical summary Summary of statistical analysis using three-way ANOVA for the effects of [CO_2_], species, and the photosynthetic type on various parameters collected for 10 grass species grown at glacial (180 μl l^–1^) and ambient (400 μl l^–1^) [CO_2_].

Parameter	Main effects (P)			Interactions (P)	
	Species	Type	[CO_2_]	[CO_2_]×species	[CO_2_]×type
Photosynthesis, A_sat_ (μmol m^–2^ s^–1^)	0.000	0.000	0.000	0.016	0.000
Conductance, g_s_ (mol m^–2^ s^–1^)	0.000	0.000	0.000	0.110	0.000
Intercellular [CO_2_], C_i_ (μl l^–1^)	0.000	0.000	0.000	0.028	0.005
PWUE [μmol (mol H_2_O)^–1^]	0.000	0.000	0.000	0.694	0.083
LMA (g m^–2^)	0.000	0.028	0.000	0.692	0.378
Leaf [N]_mass_ (mg g^–1^)	0.000	0.000	0.000	0.000	0.001
Leaf [N]_area_ (mmol m^–2^)	0.000	0.000	0.036	0.039	0.516
PNUE [mmol (mol N)^–1^ s^–1^]	0.000	0.000	0.000	0.460	0.001
Plant dry mass, PDM (g per plant)	0.000	0.000	0.000	0.000	0.000
Soluble protein (g m^–2^)	0.004	0.000	0.000	0.710	0.044
Rubisco sites (nmol m^–2^)	0.594	0.000	0.000	0.158	0.000
Rubisco/soluble protein	0.001	0.000	0.448	0.009	0.463
Rubisco-N (% leaf N)	0.006	0.000	0.407	0.230	0.000
Rubisco activity (μmol m^–2^ s^–1^)	0.000	0.000	0.000	0.004	0.000
PEPC activity (μmol m^–2^ s^–1^)	0.000	0.000	0.000	0.000	0.140
NADP-ME activity (μmol m^–2^ s^–1^)	0.000	0.000	0.340	0.048	0.461
PEP-CK activity (μmol m^–2^ s^–1^)	0.000	0.000	0.133	0.319	0.960
PEPC/Rubisco	0.000	0.000	0.003	0.068	0.579
*In vivo V*_cmax_ (μmol m^–2^ s^–1^)	0.000	0.010	0.000	0.004	0.000
*In vivo V*_pmax_ (μmol m^–2^ s^–1^)	0.000	0.000	0.000	0.000	0.004
*In vivo V*_p_/V_c_	0.000	0.010	0.000	0.000	0.001

At ambient [CO_2_], variation in *g*_s_ was unrelated to the photosynthetic type or subtype of the grasses. At glacial [CO_2_], the C_4_ species had higher *g*_s_ relative to the C_3_ and C_3_–C_4_ counterparts. Glacial [CO_2_] increased *g*_s_ to a greater extent in the C_4_ relative to the C_3_ (1.1-fold) and C_3_–C_4_ (1.3-fold) species, with NADP-ME (1.5-fold) grasses showing the greatest increase in *g*_s_ relative to the other C_4_ species (1.35-fold) (Figs 1B, 2B; Table 3; Supplementary Table S1 at *JXB* online).

At ambient [CO_2_], PWUE was higher in the C_4_ relative to the two C_3_–C_4_ and C_3_ species. At glacial [CO_2_], PWUE was highest in NAD-ME and PCK species, intermediate in NADP-ME and C_3_–C_4_, and lowest in C_3_ species. Amongst the C_4_ species, the two NAD-ME grasses had higher PWUE relative to their PCK and NADP-ME counterparts. Glacial [CO_2_] decreased PWUE in all species by an average of 55% (Figs 1C, 2C; Table 3; Supplementary Table S1 at *JXB* online).

### Leaf N use efficiency and plant dry mass

Under both [CO_2_] treatments, leaf [N]_mass_ was highest in *P. milioides* (C_3_–C_4_) and lowest in *Heteropogon contortus* (PCK). Glacial [CO_2_] enhanced leaf [N]_mass_ in all grasses except for *Panicum monticola* and *Chloris gayana* (PCK). The largest enhancement was observed in the C_3_ (51%) and NADP-ME (29%) species (Figs 1D, 2D; Table 3; Supplementary Table S1 at *JXB* online).

At ambient [CO_2_], PNUE varied 3-fold amongst the species in a manner unrelated to their photosynthetic type. Glacial [CO_2_] reduced PNUE to a lesser extent in the C_4_ (30%) relative to the C_3_ (58%) and C_3_–C_4_ (79%) species. At glacial [CO_2_], PNUE was highest in C_4_ plants (PCK >NADP-ME and NAD-ME) and lowest in C_3_ and C_3_–C_4_ plants (Figs 1E, 2E; Table 3; Supplementary Table S1 at *JXB* online).

At ambient [CO_2_], plant dry mass (PDM) was lower in the C_3_–C_4_ and NAD-ME species relative to the C_3_ and other C_4_ species. At glacial [CO_2_], the C_4_ species accumulated more biomass than their C_3_ and C_3_–C_4_ counterparts, which had similar PDM. Glacial [CO_2_] reduced PDM to a greater extent in the C_3_ (70%) and C_3_–C_4_ (42%) species relative to the C_4_ (25%) species. Amongst the C_4_ species, PDM was least and most inhibited by glacial [CO_2_] in the NAD-ME and NADP-ME grasses, respectively (Figs 1F, 2H; Table 3; Supplementary Table S1 at *JXB* online).

### Rubisco and soluble protein content

Under both [CO_2_] treatments, leaf Rubisco content was higher in *Panicum milioides* (C_3_–C_4_) relative to the other species, and in the two NAD-ME species relative to the other C_4_ grasses. At ambient [CO_2_], *P. bisulcatum* (C_3_) and NAD-ME grasses had similar Rubisco contents. Glacial [CO_2_] increased Rubisco content in *P. bisulcatum* (2.3-fold) and in three (*Astrebla lappacea*, *Panicum coloratum*, and *H. contortus*; 1.2- to 1.7-fold) of the eight C_4_ species (Tables 3, 4).

**Table 4.** Summary of leaf N, soluble protein, and Rubisco contents Ten grass species were grown at glacial (180 μl l^–1^) or ambient (400 μl l^–1^) [CO_2_]. Values are means (*n*=3–4) ±SE. Lower case letters indicate the ranking of species within each row using a multiple comparison, Tukey test. Values followed by the same letter are not significantly different at the 5% level.

Parameter	[CO_2_] (μl L^–1^)	C_3_	C_3_–C_4_	C_4_, NAD-ME		C_4_, PCK				C_4_, NADP-ME	
		*P. bisulcatum*	*P. milioides*	*A. lappacea*	*P. coloratum*	*H. contortus*	*P. monticola*	*P. maximum*	*C. gayana*	*Z. mays*	*E. frumentaceae*
LMA (g m^–2^)	180	23±9 a	21±8 a	36±13 a	46±16 a	46±22 a	48±2 a	40±14 a	16±6 a	140±11 b	
	400	61±10 a,b,c,d	25±3 a	58±3 b,c,d	51±5 a,b,c,d	76±5 d	57±5 c,d	56±4 a,b,c,d	27±8 a,b	62±3 c,d	
Leaf [N]_area_ (mmol m^–2^)	180	118±32 a,b	132±25 a,b	150±16 a,b	251±40 b	131±40 a,b	141±8 a,b	192±28 a,b	74±4 a	492±50 c	145±11 a,b
	400	98±18 a	89±15 a	154±10 a,b	170±27 a,b	144±17 a,b	190±3 b	97±11 a	91±26 a	151±2 a,b	122±17 a,b
Rubisco sites (nmol m^–2^)	180	21±6.0 b	19±6.0 b	13±0.5 a,b	15±1.0 a,b	7±0.4 a	4±0.5 a	6±0.3 a	5±0.4 a	7±0.6 a	4±0.6 a
	400	9±0.6 a,b	20±5.0 c	11±0.4 b	9±0.6 b	4±0.1 a	5±0.3 a,b	5±0.5 a	6±0.6 a,b	6±0.4 a,b	4±0.4 a
Soluble proteins (g m^–2^)	180	4.4±0.5 a,b,c	4.6±0.2 a,b,c	6.2±0.6 c	6.6±1.0 c	3.9±0.3 a,b,c	3.2±0.3 a,b	2.8±0.5 a	3.4±0.2 a,b	5.3±0.5 b,c	4.2±0.1 a,b,c
	400	2.5±0.2 a	4.0±0.3 a–d	5.0±0.5 d	4.8±0.3 c,d	2.7±0.2 a,b	3.0±0.2 a,b	3.0±0.3 a,b,c	3.2±0.3 a,b	4.1±0.2 b,c,d	3.6±0.1 a–d
Rubisco/Soluble protein	180	0.32±0.04	0.29±0.05 b,c	0.14±0.03 a,b,c	0.17±0.04 a,b,c	0.14±0.03 a,b,c	0.10±0.04 a,b	0.30±0.04c	0.10±0.04a	0.09±0.04a	0.04±0.05 a
	400	0.25±0.02 b,c	0.34±0.02 c	0.15±0.01 a	0.16±0.02 a,b	0.09±0.01 a	0.10±0.02 a	0.12±0.02 a	0.14±0.02 a	0.09±0.01 a	0.08±0.02 a
Rubisco-N (% leaf N)	180	9.8±2 a,b	14.0±2 b	5.0±1 a,b	4.2±2 a	4.8±2 a,b	2.7±1 a	2.5±2 a	5.6±2 a,b	2.9±2 a	2.1±2 a
	400	5.5±2 a	26.3±2 b	5.6±2 a	5.4±2 a	2.2±2 a	2.3±2 a	4.0±2 a	6.6±2 a	3.3±2 a	2.5±2 a

The ratio of Rubisco to soluble proteins and the proportion of leaf N invested in Rubisco (Rubisco-N) were higher in the C_3_ and C_3_–C_4_ species relative to the C_4_ species. Amongst the C_4_ species, the NADP-ME grasses tended to have the lowest leaf N or soluble protein investment in Rubisco. Glacial [CO_2_] increased Rubisco-N in the C_3_ species, reduced it in the C_3_–C_4_ species, and had little effect in the C_4_ species (Tables 3, 4).

**Fig. 3.**
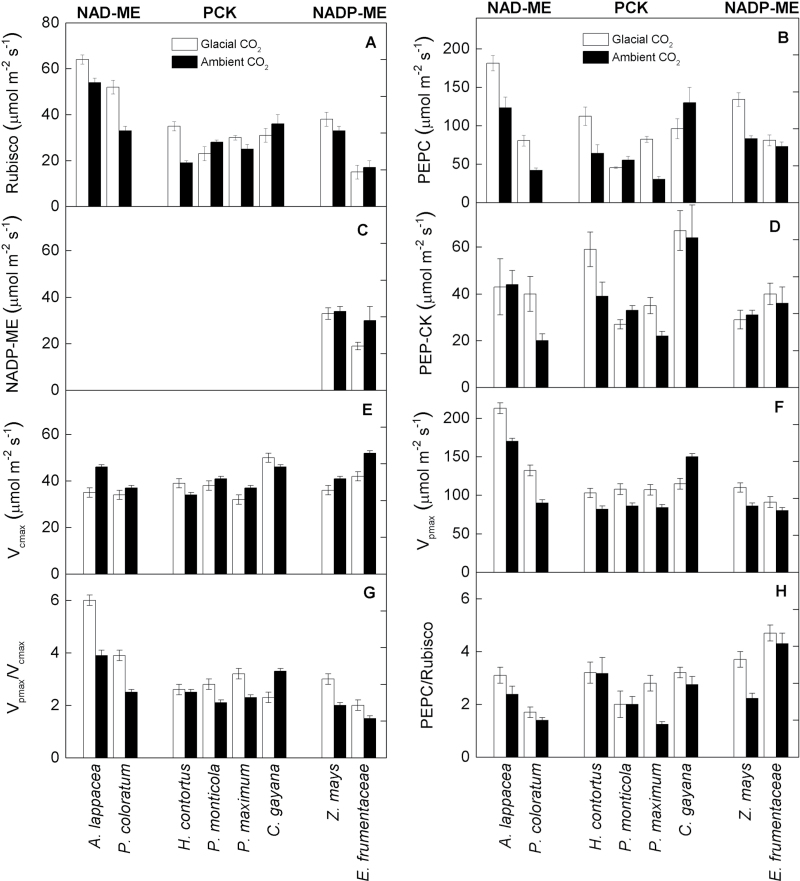
Activity of photosynthetic enzymes. Activities of Rubisco (A), PEPC (B), NADP-ME (C), PEP-CK (D), *in vivo V*_cmax_ (E), *in vivo V*_pmax_ (F), *V*_pmax_/*V*_cmax_ ratio (G), and PEPC/Rubisco activity ratio (H) of eight C_4_ grass species (NAD-ME, PCK, NADP-ME) grown at glacial (180 μl l^–1^, open columns) or ambient (400 μl l^–1^, filled columns) [CO_2_]. Values are means (*n*=3–4) ±SE.

The C_3_, C_3_–C_4_, and NAD-ME species had similar Rubisco activities, which were higher relative to the PCK and NADP-ME species. Glacial [CO_2_] significantly up-regulated Rubisco activity in the C_3_ and NAD-ME grasses only (Figs 2F, 3A, Table 3; Supplementary Table S1 at *JXB* online).

### Activity of C_4_ cycle enzymes in C_4_ grasses

At ambient [CO_2_], PEPC activity was highest in *A. lappacea* (NAD-ME) and *C. gayana* (PCK), and lowest in *P. maximum* (PCK). At glacial [CO_2_], PEPC activity was highest in *A. lappacea* and lowest in *P. monticola* (PCK). Glacial [CO_2_] stimulated PEPC activity in five out of the eight C_4_ species (Figs 2H, 3B; Table 3; Supplementary Table S1 at *JXB* online). Variations in the ratio of PEPC to Rubisco activity reflected changes in PEPC activity (Fig. 3H; Table 3; Supplementary Table S1).

In this study, only the activities of the decarboxylases NADP-ME and PEP-CK were measured. Significant activity of NADP-ME was measured in the two NADP-ME species, while marginal NADP-ME activity was detected in the two NAD-ME species and in one of the PCK species (Fig. 3C). In contrast, PEP-CK activity was ubiquitous among the C_4_ species used, with *C. gayana* showing the highest PEP-CK activity. Overall, growth [CO_2_] had no significant effect on the activity of either decarboxylase (Fig. 3C–D, Table 3; Supplementary Table S1 at *JXB* online).

The detectability of the activity of both carboxylases and decarboxylases was corroborated by immunodetection of the corresponding protein (Fig. 6). PEPC activity and protein were lacking from the C_3_ and C_3_–C_4_ species and present in all C_4_ grasses. NADP-ME activity and protein were found in two C_4_ species only. PEP-CK activity was measured in all C_4_ grasses, and the protein was readily detected in six grasses, with *A. lappacea* and *H. contortus* exhibiting weak immunoreaction with the available antibody, possibly due to divergent amino acid sequences of PEP-CK in these two species (Fig. 6).

### *In vivo* estimates of maximal Rubisco (*V*_cmax_) and PEPC activity (*V*_pmax_) in C_4_ grasses

*In vivo* estimates of *V*_cmax_ and *V*_pmax_ were calculated using the C_4_ photosynthesis model (von Caemmerer, 2000) from *A*–*C*_i_ curves measured at high light and 27 °C (Fig. 5). The variation of gas exchange-derived *V*_cmax_ between the C_4_ species was unrelated to their biochemical subtype. In contrast to its effect on *in vitro V*_cmax_ (Rubisco activity), glacial [CO_2_] reduced gas exchange *V*_cmax_ in two out of the eight C_4_ species (Fig. 3E; Table 3; Supplementary Table S1 at *JXB* online). Consequently, *in vivo* and *in vitro* estimates of *V*_cmax_ were unrelated among the C_4_ grasses (Fig. 6B). In contrast, PEPC activity was positively correlated with that of Rubisco across the C_4_ species and [CO_2_] treatments (Fig. 6A).

On average, NAD-ME species tended to have higher *V*_pmax_ and *V*_pmax_/*V*_cmax_ relative to the other C_4_ grasses, especially at glacial [CO_2_]. Glacial [CO_2_] increased *V*_pmax_ and the *V*_pmax_/*V*_cmax_ ratio in all C_4_ species, except for *C. gayana*, by an average of 25% and 19%, respectively (Fig. 3F, G; Table 3; Supplementary Table S1 at *JXB* online). Within the C_4_ species, *V*_pmax_ showed significant positive correlations with *in vitro* PEPC and Rubisco activities (Fig. 6C, D).

## Discussion

### Photosynthetic efficiency under glacial CO_2_: C_3_, C_3_–C_4_, and C_4_ pathways

In accordance with theoretical understanding, the current study revealed that photosynthetic rates (*A*_sat_) were most responsive to decreased [CO_2_] from ambient to glacial levels in C_3_ followed by C_3_–C_4_ and then C_4_ species. In addition, the C_4_ grasses had higher photosynthesis under ambient and glacial [CO_2_] relative to their C_3_ and C_3_–C_4_ counterparts (Figs 1A, 2A). Similar responses were observed for other C_3_, C_3_–C_4_, and C_4_ species exposed to 180 μl CO_2_ l^–1^ and 380 μl CO_2_ l^–1^ (Ward *et al.*, 1999; Cunniff *et al.*, 2010; Pinto *et al.*, 2011; Vogan and Sage, 2012).

Stomatal conductance was greater at glacial [CO_2_] compared with ambient [CO_2_] in all species, but in particular was higher in C_4_ species relative to the C_3_ and C_3_–C_4_ species (Figs 1B, 2B). Huxman and Monson (2003) found that *g*_s_ was more sensitive to changing *C*_i_ in C_4_ relative to C_3_ and C_3_–C_4_ *Flaveria* species. Recently, Vogan and Sage (2011) presented evidence of changed *C*_i_ sensitivity for *g*_*s*_ in *Flaveria* species during their evolutionary transition from C_3_ to C_4_ photosynthesis. In contrast, Morison and Gifford (1983) observed little difference in stomatal sensitivity to short-term changes of [CO_2_] or vapour pressure deficit between two C_3_ and two C_4_ grasses. Growth at low [CO_2_] may cause acclimation of the stomatal response that is not necessarily captured during short-term gas exchange measurements. However, a number of studies found no evidence of differential stomatal acclimation between C_3_ and C_4_ plants (Cunniff *et al.*, 2010; Vogan and Sage, 2012). Hence, there does not seem to be a consensus regarding the relative stomatal sensitivity to short- or long-term changes in [CO_2_] between C_3_ and C_4_ plants, which remains an area worthy of further investigation.

Despite having larger *g*_s_ at glacial [CO_2_], C_4_ species maintained greater PWUE than C_3_–C_4_ and C_3_ species as a result of higher photosynthetic rates in C_4_ plants (Fig. 1). Improved PWUE is one of the most consistently reported advantages of C_4_ species (Long, 1999; Taylor *et al.*, 2010). Higher PWUE in the C_3_–C_4_ species relative to the C_3_ species under both growth [CO_2_] confirmed that the photorespiratory pump of the intermediate pathway confers greater water use efficiency relative to the C_3_ pathway (Pinto *et al.*, 2011; Vogan and Sage, 2011), thereby achieving PWUE similar to the C_4_, NADP-ME pathway under glacial [CO_2_] (Fig. 1C).

**Fig. 4.**
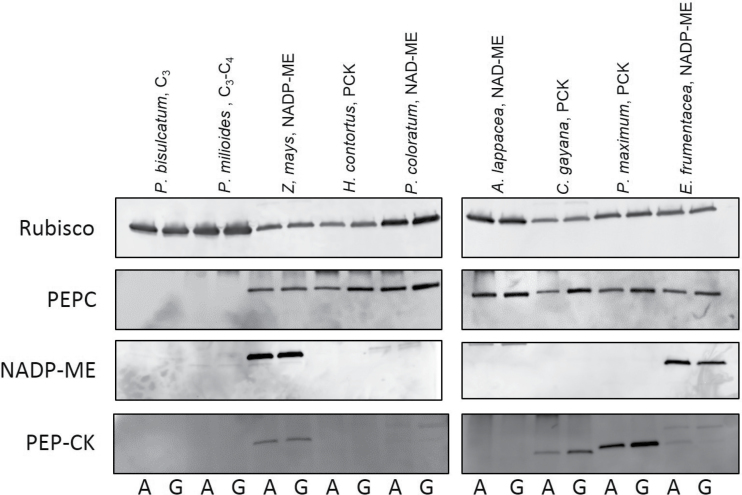
Immunoblot analyses of photosynthetic enzymes. Examples of immunoblot analysis for the photosynthetic proteins Rubisco (A), PEPC (B), NADP-ME (C), and PEP-CK (D) extracted from leaves of selected grass species grown at glacial (180 μl l^–1^, G) or ambient (400 μl l^–1^, A) [CO_2_].

Higher PNUE in C_4_ relative to C_3_ plants under ambient [CO_2_] is well established (Brown, 1978; Long, 1999; Taylor *et al.*, 2010). In this study, these differences were maintained under glacial [CO_2_] as a result of higher photosynthetic rates and lower leaf [N] in the C_4_ relative to the C_3_ and C_3_–C_4_ species (Fig. 1). The C_3_–C_4_ species had no PNUE advantage over the C_3_ species, mainly due to the higher leaf [N] and Rubisco-N of the intermediate species (Table 4). In contrast, intermediate *Flaveria* species maintained higher photosynthesis and PNUE relative to C_3_ congeners at ambient and glacial [CO_2_] (Vogan and Sage, 2012).

Growth of *P. bisulcatum* (C_3_) at glacial [CO_2_] increased Rubisco activity and *g*_s_ to improve photosynthetic capacity and CO_2_ supply, respectively (Tissue *et al.*, 1995; Gesch *et al.*, 2000; Anderson *et al.*, 2001). These commonly reported responses represent significant N and water costs for C_3_ plants at glacial [CO_2_], thus reducing their PWUE and PNUE. The additional resource requirements at low [CO_2_] may have contributed to the more pronounced reduction in plant biomass in C_3_ relative to C_4_ plants observed in this study (Fig. 2F) as in others (Ward *et al.*, 1999; Cunniff *et al.*, 2010; Ripley *et al.*, 2013). Consequently, low WUE and NUE of C_3_ photosynthesis at low [CO_2_] may have favoured the evolution of C_4_ phototosynthesis.

### Photosynthetic efficiency under glacial CO_2_: the C_4_ subtypes

Results obtained in this study at glacial [CO_2_] largely confirmed previously reported differences in photosynthetic efficiency among the C_4_ subtypes at ambient [CO_2_], and revealed a number of insights into the physiology of C_4_ subtypes, as discussed below.

First, there were no subtype differences in photosynthetic rates or their sensitivity to decreased growth [CO_2_]. These results constitute new evidence that there are no discernible differences in the efficiency of the CCM operating in the three C_4_ subtypes, despite their diverse leaf biochemistry and anatomy. This conclusion is supported by the findings that CO_2_ leakiness out of the bundle sheath (a surrogate measure of CCM efficiency) is similar among C_4_ grasses with different subtypes (Henderson *et al.*, 1992; Cousins *et al.*, 2008).

Secondly, NAD-ME species had lower *g*_s_ and higher PWUE relative to NADP-ME and PCK counterparts at glacial [CO_2_]. Moreover, *g*_s_ was less affected by glacial [CO_2_] in NAD-ME than in NADP-ME and PCK grasses (Fig. 2). Previous studies demonstrated that photosynthetic activity was less sensitive to water deficit, and leaf traits were better suited for arid habitats in an NAD-ME relative to an NADP-ME and a PCK grass (Carmo-Silva *et al.*, 2007, 2009). In another study, Ghannoum *et al.* (2002) showed that NAD-ME grasses increased their whole-plant WUE to a greater extent than their NADP-ME counterparts under water stress. Taken together, these findings are consistent with the observation that grasses with the NAD-ME subtype predominate in more arid regions relative to the other two C_4_ subtypes (Hattersley, 1992; Taub, 2000).

Thirdly, NADP-ME grasses showed the greatest increase of leaf [N]_mass_, which may be linked to their stomatal response in that the correlation between N uptake (proxy leaf [N]) and mass flow of soil water through the transpiration stream (proxy *g*_s_) is commonly reported in plants grown under different atmospheric [CO_2_] (Conroy and Hocking, 1993; McDonald *et al.*, 2002; Sherwin *et al.*, 2013).

Fourthly, NAD-ME grasses showed the lowest biomass reduction in response to decreased growth [CO_2_] relative to the PCK and NADP-ME species. NAD-ME grasses also had lower plant biomass relative to the other C_4_ species at both growth [CO_2_]. Studies conducted at elevated [CO_2_] have shown that growth response to high [CO_2_] decreases with decreasing growth potential (Poorter, 1993; Ziska and Bunce, 1997). Extrapolating these findings to low [CO_2_] suggests that the lower growth response to glacial [CO_2_] in NAD-ME plants may be related to their smaller biomass accumulation relative to the other, larger C_4_ species.

### Photosynthetic enzymes under glacial CO_2_

Generally, growth at low [CO_2_] leads to increased photosynthetic capacity, *g*_s_, and leaf [N] in C_3_ plants (Dippery *et al.*, 1995; Ward *et al.*, 1999; Anderson *et al.*, 2001; Cunniff *et al.*, 2010; Gerhart and Ward, 2010; Ripley *et al.*, 2013). Accordingly, *P. bisulcatum* (C_3_) exhibited increased leaf proteins, including Rubisco at glacial [CO_2_] (Fig. 2; Table 4). *Panicum milioides* (C_3_–C_4_) did not up-regulate Rubisco content at glacial [CO_2_], possibly due to the high leaf [N] and Rubisco-N in this species; a consequence of the high N costs of operating two Calvin cycles in the mesophyll and bundle sheath cells (Monson, 1989; Monson and Rawsthorne, 2000).

**Fig. 5.**
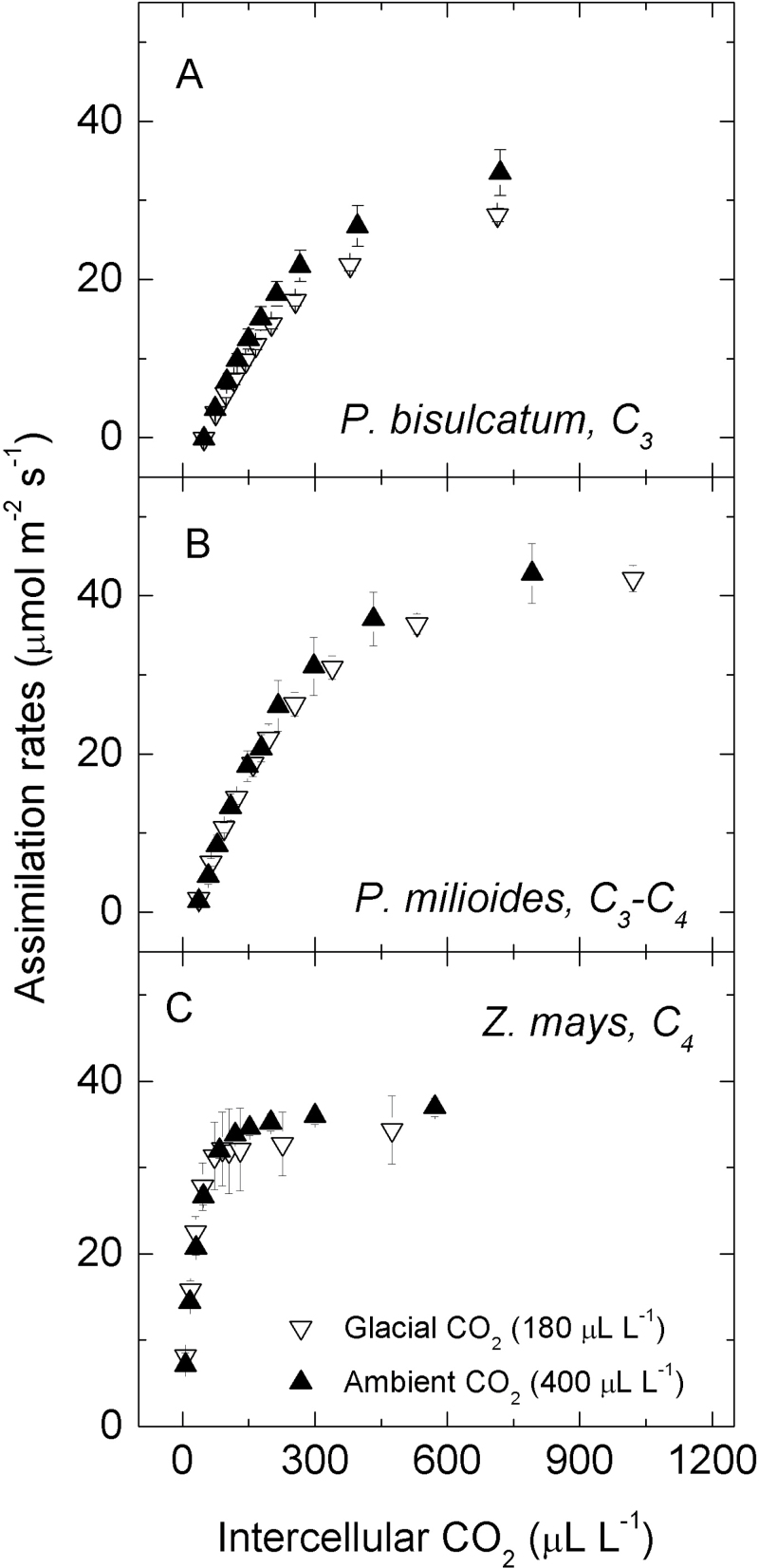
Responses of CO_2_ assimilation rate to increasing intercellular [CO_2_]. Examples of *A*–*C*_i_ curves measured in C_3_, C_3_–C_4_, and C_4_ species grown at glacial (180 μl l^–1^, inverted open triangles) or ambient (400 μl l^–1^, filled triangles) [CO_2_]. Values represent the means ±SE of three replicates.

**Fig. 6.**
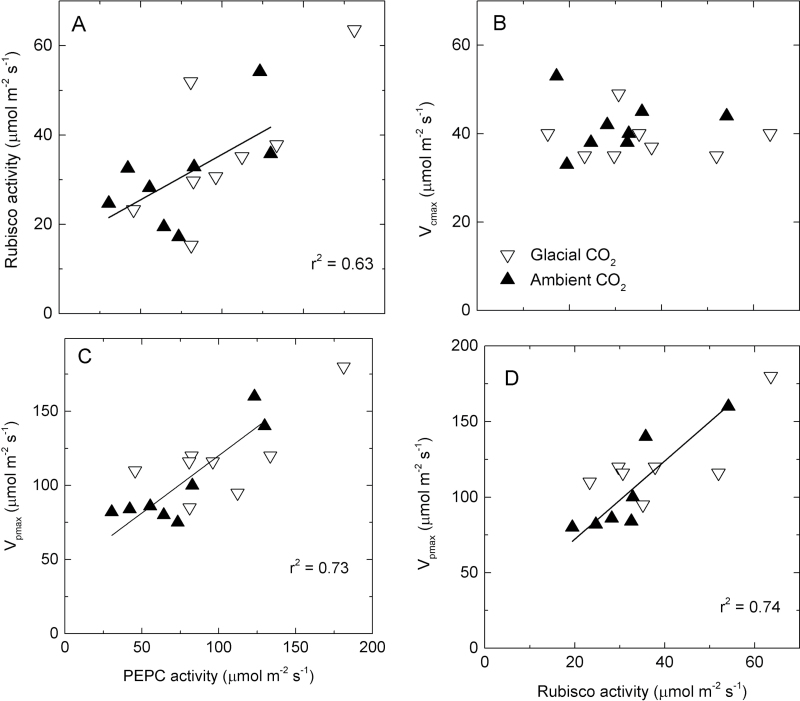
Relationships between the *in vitro* and *in vivo* estimates of Rubisco and PEPC activities in eight C_4_ grass species. Values are means for each species grown at glacial (180 μl l^–1^, inverted open triangles) or ambient (400 μl l^–1^, filled triangles) [CO_2_]. Solid lines represent linear regressions of all data points. Original data are shown in Supplementary Table S1 at *JXB* online.

The operation of Rubisco under elevated [CO_2_] in the bundle sheath, the multiplicity of metabolic cycles and cells involved in C_4_ photosynthesis, and the complexity of its regulation thwart the task of predicting how C_4_ photosynthesis will acclimate to growth at low [CO_2_]. Measurements of photosynthetic rates under growth [CO_2_] (*A*_sat_) indicated that photosynthesis in the C_4_ grasses was CO_2_ limited at glacial [CO_2_], albeit to a lesser extent than C_3_ and C_3_–C_4_ counterparts (Fig. 2A). This may explain the significant up-regulation of the two carboxylases, Rubisco and PEPC, which was observed in a number of the C_4_ grasses (Figs 3–6). Generally, the activities of Rubisco and PEPC changed in concert, a reflection of the fine balance operating between these two enzymes which modulate the pace of the C_3_ and C_4_ cycles during C_4_ photosynthesis, respectively (von Caemmerer and Furbank, 2003). There is strong evidence showing that CO_2_ delivery into the bundle sheath and fixation in the mesophyll are tightly regulated, as indicated by the constancy of leakiness (a measure of CO_2_ fixed by PEPC but not Rubisco, subsequently leaking back from the bundle sheath) under a wide range of environmental conditions (Henderson *et al.*, 1992; Cousins *et al.*, 2008). Nevertheless, the PEPC/Rubisco ratio increased at glacial [CO_2_] in two C_4_ species (Fig. 3H). Increasing PEPC/Rubisco via transgenic transformation in *Flaveria bidentis* led to increased leakiness, an indication of reduced efficiency of the C_4_ mechanism (von Caemmerer *et al.*, 1997b). In the current study, *V*_pmax_ and PEPC activity were linearly correlated, while *V*_cmax_ and Rubisco activity showed no correlation (Fig. 5). Reconciling the *in vivo* and *in vitro* estimates of Rubisco and PEPC activity will require greater knowledge about bundle sheath cell wall conductance and [CO_2_] than is currently available (von Caemmerer *et al.*, 1997a; von Caemmerer and Furbank, 2003).

The activities of the two measured decarboxylases were not affected by growth [CO_2_], possibly reflecting the low control that decarboxylases exert on the photosynthetic flux. Pengelly *et al.* (2012) reported that NADP-ME activity in transgenic *F. bidentis* can be halved without affecting photosynthetic rates or growth. Accordingly, the rate of the decarboxylases measured at ambient [CO_2_] may be sufficient under glacial [CO_2_], where Rubisco and PEPC activities were up-regulated in a number of C_4_ species. Although PEPC and NADP-ME have significant effects on the efficiency of the C_4_ pathway as evidenced by changes in leakiness, Rubisco retains a high control of metabolic flux in C_4_ leaves (Furbank *et al.*, 1997; von Caemmerer *et al.*, 1997b; Pengelly *et al.*, 2012).

It is worth noting that PEP-CK activity and, to a lesser extent, PEP-CK protein were ubiquitously detected in the C_4_ species used in this study. Significant PEP-CK activity in C_4_ grasses and eudicots of the NADP-ME and NAD-ME subtypes has been previously reported (Walker *et al.*, 1997; Wingler *et al.*, 1999; Carmo-Silva *et al.*, 2008; Muhaidat and McKown, 2013). These findings challenge the classical view of the C_4_ subtypes, where a single decarboxylase dominates (Hatch, 1987; Furbank, 2011). Recent studies have postulated a role for PEP-CK as a second decarboxylase in maize that serves to match ATP and NADPH demand in bundle sheath and mesophyll cells under different light environments (Bellasio and Griffiths, 2013). The full physiological significance of PEP-CK in a wider range of C_4_ grasses and environments is yet to be elucidated.

## Conclusions

Various photosynthetic responses, including increased leaf Rubisco, nitrogen, and *g*_s_, were observed in response to growth at glacial [CO_2_]. Nevertheless, the operation of a CCM ensured that PWUE and PNUE remained higher in C_4_ species relative to C_3_ and C_3_–C_4_ species, while the photorespiration pump ensured higher PWUE in the C_3_–C_4_ relative to the C_3_ species. Greater resource use efficiency promotes cheaper biomass construction costs, and hence reduces productivity losses at low [CO_2_]. Accordingly, high resource use efficiency may have constituted a key evolutionary advantage for the transition from C_3_ to C_4_ photosynthesis under low [CO_2_] (Cerling *et al.*, 1998; Sage, 2004). Results obtained in this study support the notion that Rubisco and PEPC, rather than the decarboxylases, modulate the response to glacial [CO_2_] for C_4_ grasses with different biochemical subtypes.

## Supplementary data

Supplementary data are available at *JXB* online

Table S1. Summary of leaf gas exchange, resource use efficiency, and activity of photosynthetic enzymes.

Supplementary Data
