# Supplementary material for: Photosynthesis of C3, C3–C4, and C4 grasses at glacial CO2
Source: J Exp Bot. 2014 Apr 10;65(13):3669–81. doi: 10.1093/jxb/eru155 (PMC4085965; doi:10.1093/jxb/eru155)
Supplement: Supplementary Data [file supp_65_13_3669__index.html]

Photosynthesis of C3, C3–C4, and C4 grasses at glacial CO2 — Supplementary Data 

# Photosynthesis of C3, C3–C4, and C4 grasses at glacial CO2

## Supplementary Data

Data files

**Files in this Data Supplement:**

- Supplementary Data - Supplementary Data
