# Supplementary material for: Photosynthesis of C3, C3–C4, and C4 grasses at glacial CO2
Source: J Exp Bot. 2014 Apr 10;65(13):3669–81. doi: 10.1093/jxb/eru155 (PMC4085965; doi:10.1093/jxb/eru155)
Supplement: Supplementary Data [file supp_eru155_116947_Supplementary_Table_S1.pdf]

Supplementary Table S1. Summary of leaf gas exchange, resource use efficiency and activity of photosynthetic enzymes.

Ten grass species were grown at glacial (180 µl L^{-1}) or ambient (400 µl L^{-1}) [CO_2]. Values are means (n = 3-4) ± SE. Superscripts indicate the ranking of species within each raw using a multiple-comparison, Tukey test. Values followed by the same letter are not significantly different at the 5% level. n/d = not detected.

**Parameter**	**[CO_2] (µl L^{-1})**	**C_3**	**C_3-C_4**	**C_4, NAD-ME**		**C_4, PCK**				**C_4, NADP-ME**	
		P. bisulcatum	*P. milioides*	*A. lappacea*	*P. coloratum*	*H. contortus*	*P. monticola*	*P. maximum*	*C. gayana*	*Z. mays*	*E. frumentaceae*
**Spot leaf gas exchange**											
A_{sat}	180	7 ± 1^{a}	8 ± 1^{a}	25 ± 0^{c}	23 ± 1^{c}	16 ± 1^{b}	25 ± 0^{c}	23 ± 0^{c}	32 ± 1^{d}	27 ± 0^{cd}	27 ± 2^{cd}
(µmol m^{-2} s^{-1})	400	17 ± 1^{a}	22 ± 3^{ab}	39 ± 0^{ef}	29 ± 0^{bcd}	26 ± 0^{b}	34 ± 0^{bc}	27 ± 0^{bc}	42 ± 0^{f}	33 ± 1^{cde}	38 ± 1^{ef}
g_s	180	0.24 ±0.04^{ab}	0.16 ±0.02^{a}	0.29±0.01^{abc}	0.32±0.01^{abc}	0.19 ±0.01^{a}	0.43±0.02^{b-e}	0.35±0.01^{a-d}	0.53±0.08^{de}	0.48±0.02^{cde}	0.64 ±0.09^{e}
(mol m^{-2} s^{-1})	400	0.21±0.02^{abc}	0.24±0.04^{bcd}	0.21±0.01^{abc}	0.19±0.01^{abc}	0.15±0.01^{a}	0.26±0.01^{cd}	0.17±0.01^{ab}	0.33±0.02^{de}	0.21 ±0.01^{abc}	0.35 ±0.04^{e}
C_i	180	115 ± 4^{e}	93 ± 4^{de}	21 ± 1^{a}	36 ± 1^{ab}	35 ± 8^{ab}	47 ± 3^{bc}	41 ± 4^{ab}	55±12^{bc}	44 ± 4^{ab}	70 ± 5^{cd}
(µl L^{-1})	400	231 ± 3^{e}	210 ± 19^{de}	57 ± 9^{a}	109 ±24^{abc}	93 ±15^{abc}	137±5^{bcd}	103 ±12^{abc}	159±13^{cde}	80 ± 19^{ab}	162 ± 23^{cde}
**Analysis of A-C_i curves**											
in vivo V_{cmax}	180	83 ± 5		35 ± 2^{ab}	34 ± 2^{ab}	39 ± 2^{ab}	38 ± 2^{ab}	32 ± 2^{a}	50 ± 2^{c}	36 ± 2^{ab}	42± 2^{bc}
(µmol m^{-2} s^{-1})	400	104 ± 8		46 ± 1^{bc}	37 ± 1^{a}	34 ± 1^{a}	41 ± 1^{ab}	37 ± 1^{a}	46 ± 1^{bc}	41 ± 1^{ab}	52± 1^{c}
in vivo V_{pmax}	180			213 ± 7^{c}	132 ± 7^{b}	103 ± 6^{ab}	108 ± 7^{ab}	107 ± 7^{ab}	115 ± 7^{ab}	110 ± 6^{ab}	91 ± 7^{a}
(µmol m^{-2} s^{-1})	400			170 ± 4^{c}	90 ± 4^{a}	82 ± 4^{a}	86 ± 4^{a}	84 ± 4^{a}	150 ± 4^{b}	86 ± 4^{a}	80 ± 4^{a}
V_p/V_c	180			6 ± 0.2^{f}	3.9 ±0.2^{e}	2.6 ±0.2^{ab}	2.8 ± 0.2^{bc}	3.2 ±0.2^{ab}	2.3 ±0.2^{ab}	3 ± .2^{de}	2 ± 0.2^{a}
	400			3.7 ± 0.1^{c}	2.5 ±0.1^{b}	2.5 ±0.1^{b}	2.1 ± 0.1^{ad}	2.3 ±0.1^{b}	3.3 ±0.1^{c}	2 ± 0.1^{ab}	1.5 ± 0.1^{a}
**Resource use efficiency**											
PWUE	180	32 ± 3^{a}	48 ± 2^{ab}	84 ± 1^{d}	73 ± 5^{cd}	83 ± 5^{d}	59 ± 2^{bc}	65 ± 2^{bcd}	63 ± 7^{bc}	55±2^{bc}	45 ± 5^{ab}
(µmol mol^{-1})	400	83 ± 3^{a}	94 ± 12^{ab}	189 ± 6^{d}	158 ± 15^{cd}	177 ± 10^{d}	131 ± 2^{bc}	159 ± 9^{cd}	126 ± 8^{bc}	160±11^{cd}	113 ± 14^{ab}
PNUE	180	0.06 ±0.02^{ab}	0.06±0.01^{a}	0.16±0.02^{abc}	0.10±0.02^{abc}	0.15±0.05^{abc}	0.18±0.01^{bc}	0.13±0.02^{abc}	0.43±0.02^{d}	0.06±0.01^{a}	0.19±0.02^{c}
(mmol mol^{-1} s^{-1})	400	0.20±0.05^{ab}	0.28±0.08^{ab}	0.25 ±0.0[illegible]^{ab}	0.18 ±0.02^{a}	0.18 ±0.02^{a}	0.18±0.01^{a}	0.28±0.04^{bc}	0.59 ±0.02^{b}	0.22±0.01^{ab}	0.32 ±0.04^{ab}
PDM	180	12 ± 1^{ab}	10 ± 1^{a}	30 ± 1^{cd}	18 ± 1^{b}	31 ± 1^{cd}	36 ± 1^{de}	27 ± 1^{c}	38 ± 1^{e}	34 ± 1^{de}	25 ± 1^{c}
(g $plant^{-1}$)	400	41 ± 1^{bc}	15 ± 1^{a}	35 ± 1^{b}	15 ± 1^{a}	42 ± 1^{bc}	33 ± 1^{b}	45 ± 1^{cd}	53 ± 1^{d}	86 ± 1^{e}	47±1^{cd}
**Enzyme activity**											
Rubisco activity	180	55 ± 15^{c}	43±14^{abc}	64 ± 2^{bc}	52 ± 3^{bc}	35 ± 2^{abc}	23 ± 3^{ab}	30 ± 1^{abc}	31 ± 3^{ab}	38 ± 3^{abc}	15 ± 3^{a}
(µmol m^{-2} s^{-1})	400	23 ± 2^{abc}	44 ± 11^{de}	54 ± 2^{e}	33 ± 2^{cd}	19 ± 1^{ab}	28 ± 1^{abcd}	25 ± 2^{abc}	36 ± 4^{bcd}	33 ± 2^{abcd}	17 ±2^{a}

PEPC activity	180			181 ± 10^{d}	81 ± 7^{ab}	112 ± 12^{bc}	46 ± 1^{a}	82 ± 4^{ab}	96 ± 13^{bc}	134 ± 9^{c}	81 ± 7^{ab}
(μmol m^{-2} s^{-1})	400			123 ± 14^{cd}	42 ± 3^{ab}	64 ± 11^{ab}	55 ± 5^{ab}	30 ± 4^{a}	130 ± 20^{d}	83 ± 4^{bc}	73 ± 6^{ab}
PEPC/Rubisco	180			3.1± 0.3^{cd}	1.7 ± 0.2^{a}	3.2 ± 0.4^{a-d}	2.0± 0.5^{ab}	2.8± 0.3^{abc}	3.2± 0.2^{bcd}	3.7± 0.3^{cd}	4.7 ± 0.3^{d}
	400			2.4± 0.3^{bc}	1.4 ±0.1^{a}	3.2 ± 0.6^{bc}	2.0 ±0.3^{ab}	1.2 ± 0.1^{a}	2.8 ±0.3^{bc}	2.2± 0.2^{ab}	4.3 ± 0.4^{c}
NADP-ME activity	180			1 ± 0.3	3 ± 0.2	3 ± 0.2	n/d	n/d	n/d	33 ± 3	19 ± 2
(μmol m^{-2} s^{-1})	400			1 ± 0.2	1 ± 0.2	2 ± 0.2	n/d	n/d	n/d	34 ± 2	30 ± 6
PEP-CK activity	180			43 ± 12 ^{b}	40 ± 8 ^{b}	59 ± 7.4^{bc}	27 ± 2^{a}	35 ± 4^{b}	67 ± 9 ^{c}	29 ± 4 ^{a}	40 ± 5 ^{b}
(μmol m^{-2} s^{-1})	400			44 ± 6 ^{c}	20 ± 3^{a}	39 ± 6^{b}	33 ± 2^{b}	22 ± 2^{a}	64 ± 14 ^{d}	31 ± 2 ^{b}	36 ± 7 ^{b}
